# A prospective randomized half-body study: 308 nm LED light vs. 308 nm excimer laser for localized psoriasis

**DOI:** 10.3389/fmed.2023.1275912

**Published:** 2023-11-06

**Authors:** Yingyuan Yu, Jiajing Lu, Yi Zhang, Yuling Shi

**Affiliations:** ^1^Shanghai Skin Disease Hospital, School of Medicine, Tongji University, Shanghai, China; ^2^Institute of Psoriasis, School of Medicine, Tongji University, Shanghai, China

**Keywords:** psoriasis, phototherapy, excimer, light emitting diode, dermatoscopy

## Abstract

**Background:**

Psoriasis is a chronic skin disease affecting approximately 3.2% of the population. The 308 nm light emitting diode (LED) is a novel, portable, and cost-effective light source, may have potential in the treatment of localized psoriasis patients in a home setting.

**Objective:**

To compare the clinical and dermoscopic responses in localized psoriatic patients undergoing localized phototherapy with 308 nm LED light and excimer laser.

**Methods:**

Twenty-two patients with mild-to-moderate psoriasis and symmetrical skin lesions were included in this prospective, randomized, left-to-right body trial. The target lesions were randomly treated with either LED light or excimer laser twice a week for 12 weeks. The responses were evaluated by the local psoriasis severity index (LPSI) scores, and dermoscopic features of the target lesions were examined and analyzed.

**Results:**

Out of the 22 included psoriasis patients, 10 successfully completed the 12 weeks study. Both treatment sides showed similar clinical improvement in terms of clinical response, as evidenced by a LPSI 50 rate of 70% on the LED side and 80% on the excimer side, *p* > 0.05. Furthermore, the dermoscopic features also exhibited comparable improvement.

**Conclusion:**

The efficacy and safety of 308 nm LED light therapy are comparable to 308 nm excimer laser therapy. Moreover, given the portability and cost-effectiveness of 308 nm LED light, it holds great promise as a home phototherapy in the treatment of psoriasis.

## 1. Introduction

Psoriasis is a chronic condition characterized by erythematous, scaly patches on the body. It affects approximately 3.2% of the population (1). Targeted 308 nm excimer light therapy is well-established for psoriasis, particularly for localized psoriasis management. 308 nm excimer light precisely target the affected area, ensuring therapeutic effectiveness while minimizing exposure to unaffected healthy skin (2). In the case of localized psoriasis vulgaris, the effectiveness of the 308 nm excimer laser has been demonstrated to surpass that of narrowband ultraviolet B (NB-UVB) therapy (3). The 308 nm devices predominantly utilized in hospitals include excimer lasers and excimer lights, with the latter being more cost-effective than excimer lasers. However, both of them are more expensive than light emitting diode (LED). Excimer laser devices are not suitable for home treatment and are typically limited to hospital settings. The cost of excimer light is lower than excimer laser, however, its cost is several times higher than that of LED light, which can still pose a significant financial burden for home use. In addition, LED light has a longer lifespan, and its lower radiation intensity can bring greater treatment comfort compared to excimer laser devices, further enhancing the long-term cost-effectiveness and comfort of treatment (4). Phototherapy is one of the first-line treatment options recommended (1), however, undergoing phototherapy at a medical facility can be burdensome in terms of time, cost, and convenience. Patients are required to attend hospital frequently, bear the financial copayments of transportation, and experience lost wages due to the need for ongoing treatment sessions (5). In particular, for patients just with localized mild to moderate psoriasis, frequent hospital visits for treatment result in a low cost-effectiveness ratio. The 308 nm LED represents an economical, portable phototherapy device which can be conveniently used at home, sharing a similar spectral profile with the 308 nm excimer laser. The 308 nm LED exhibits comparable skin penetration depth and operates through similar mechanisms. In a 2017 review, it was mentioned that LED can be an alternative light source advantageous and feasible, especially when their intensity level is sufficiently high to effectively treat skin diseases (6). A retrospective study has examined the effectiveness and safety of 308 nm LED treatment for vitiligo, demonstrated its practical application in vitiligo treatment (7). The 308 nm LED light source may be more suitable for home treatment of localized psoriasis patients. However, currently, there is a lack of research on the efficacy and safety of LED-308 nm devices specifically designed for home-based treatment. Therefore, we conducted a comparative analysis of the efficacy and safety between 308 nm LED light and 308 nm excimer laser in the treatment of local plaque psoriasis.

## 2. Materials and methods

### 2.1. Study design

This study was a prospective, single-center, randomized, left-right comparative trial. The aim of the study was to evaluate the efficacy and safety of two different 308 nm light sources: the excimer 308 nm laser and the LED 308 nm light. A total of 22 patients with mild to moderate plaque psoriasis were enrolled in this study at Shanghai Skin Diseases Hospital from March 2021 to December 2021. The eligibility criteria for inclusion were established in Supplementary Table S1. Patients with an equal distribution of left and right discrimination were selected, and each patient participating in the study selected two symmetrical psoriatic lesions with similar thickness and area as target lesions. The treatment for these lesions was assigned randomly, utilizing the aforementioned devices. The study was conducted in accordance with the Declaration of Helsinki, and approved by the Ethics Committee of Shanghai Skin Disease Hospital (approval #2020-32). Informed consent was obtained from all participants prior to enrollment.

### 2.2. Devices and treatment protocol

The 308 nm LED light phototherapy device (SQ1, SIGMA High-tech Co., Shanghai, China) (Supplementary Figure S1) was utilized as the experimental device in this study, while the 308 nm excimer laser phototherapy device (ML-7085, MIRACLE, Wuhan, China) was used as the control. The peak wavelength of the two devices was both 308 ± 2 nm. The irradiation intensity of 308 nm LED light device was 23 mW/cm^2^, while the irradiation intensity of 308 nm excimer laser device was 50 mW/cm^2^. The largest spot size with this 308 nm LED light device was 900 mm^2^ (30 × 30 mm), and 3,000 mm^2^ (50 × 60 mm) for this 308 nm excimer laser device. The two devices were calibrated every 3 months to ensure stable light output. The treatment with both devices was conducted in hospital and administered by physicians. The treatment protocols adhered to the recommendations outlined in the American Academy of Dermatology (AAD) Guidelines (Supplementary Table S2) (1), and administered twice a week for a maximum of 24 sessions. Initial dosages were calculated based on individual skin types and plaque thickness. If there was no erythema reaction, the dose was increased by 25%. If the erythema reaction was slight, the dose was increased by 15%. If the erythema reaction was mild-to-moderate, the dose was maintained. If significant improvement with plaque thinning or reduced scaliness or pigmentation occurred, the irradiation dose was reduced by 10% or maintained. If moderate/severe erythema (with or without blistering), treatment dose was reduced by 25%. During the study period, the use of topical or systemic therapy for the treatment of psoriasis, except for emollients and antihistamines, was not allowed.

### 2.3. Assessments

1. Efficacy assessments. The local psoriasis severity index (LPSI) score was calculated by monitoring erythema, infiltration, and scales and then scoring each of these parameters with a score of 0 to 4 (0: not present; 1: mild, 2: moderate; 3: severe; 4: very severe). The LPSI score was assessed by two dermatologists at baseline and after 12 weeks treatment. Poor treatment efficacy was defined as reduction of <50% in LPSI from baseline. Dermoscopic evaluation was used to assess specific dermoscopic features, including background erythema, scales, hemorrhagic spots, and hemorrhagic crust to further assess the treatment response.
2. Safety assessments. All adverse events associated with the treatments were recorded during the treatment.

### 2.4. Statistical analysis

Data regarding clinical response and safety outcomes were collected at specified intervals throughout the treatment period. Continuous data were compared using t-test analysis, while Fisher’s exact test was employed for categorical data. A *p*-value <0.05 was considered statistically significant. All data analysis was performed using Prism Version 9.5.0.

## 3. Results

### 3.1. Patient distribution

Due to the frequent need for patients to visit the hospital for treatment, which imposed a burden on the patients, we decided to discontinue further enrollment despite not reaching the target sample size. In total, 10 out of 22 patients completed 12 weeks of treatment. Among the patients who did not complete the treatment, 5 out of 12 discontinued due to poor efficacy. Besides, 5 out of 12 patients showed improvement in their skin lesions, but they chose not to continue with the treatment. One out of the 12 discontinued due to erythema with pain on both sides. The erythema resolved within 3 days. Lastly, one patient out of the 12 discontinued for personal reasons. The mean number of treatments was 16.0.

### 3.2. Clinical efficacy

**Figure 1:**
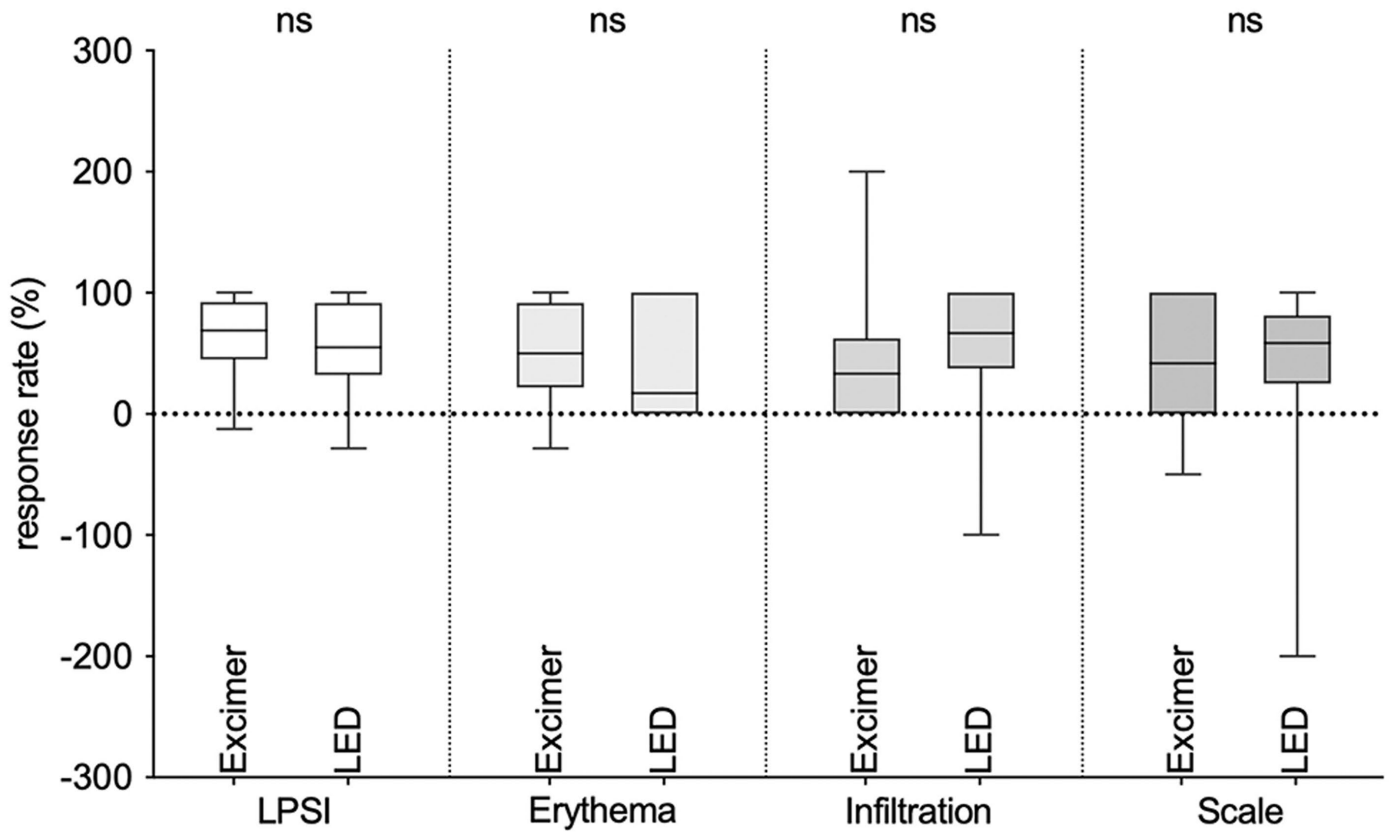
Changes in the local psoriasis severity index (LPSI) score and erythema, infiltration, and scales scores before and after treatment of 308 nm LED light versus 308 nm excimer laser.

**Figure 2:**
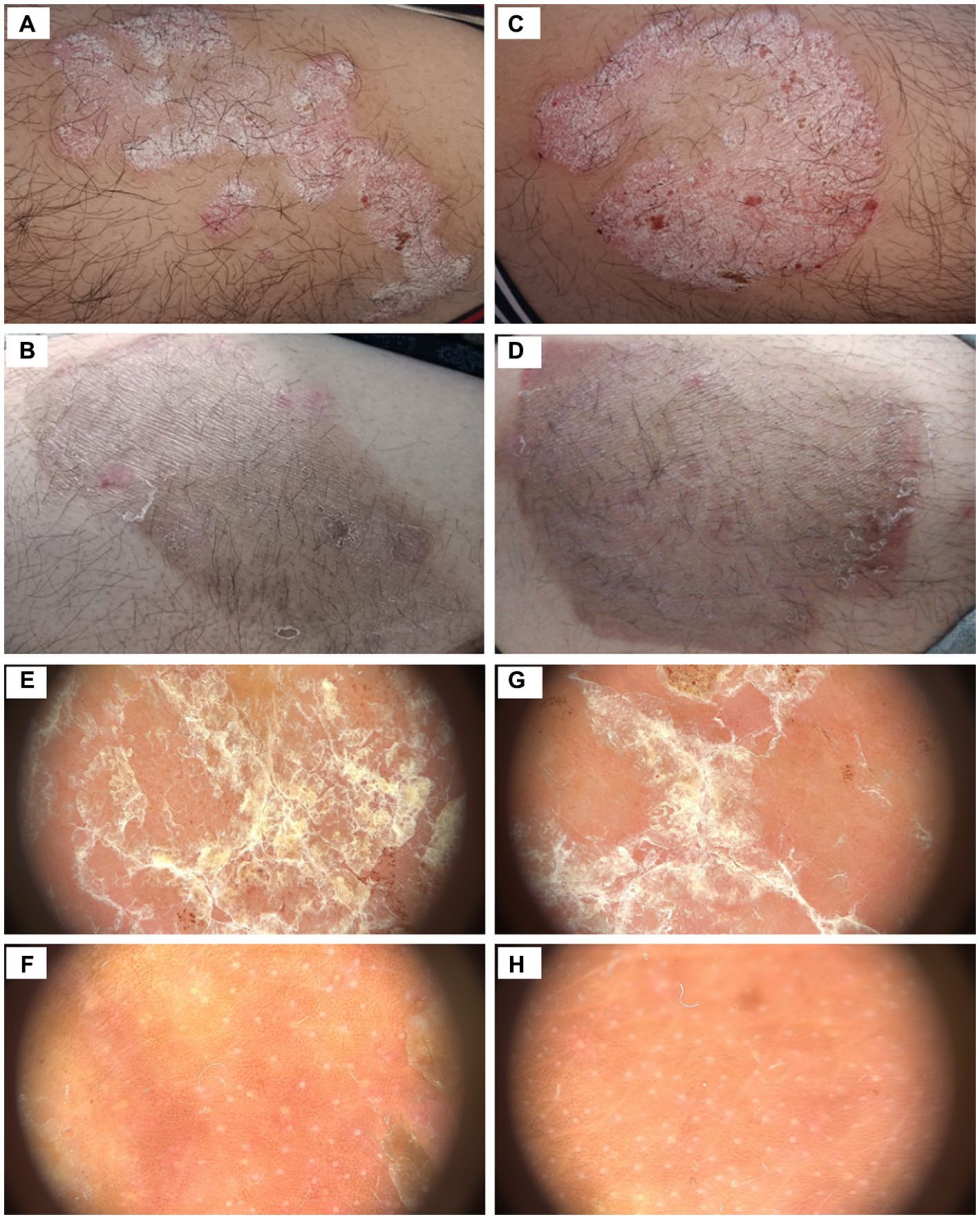
Clinical pictures and dermatoscopy examination of target psoriatic plaques of a patient, **(A)** before 308 nm LED phototherapy, **(B)** after 308 nm LED phototherapy, **(C)** before 308 nm excimer laser phototherapy, **(D)** after 308 nm excimer laser phototherapy, **(E)** before 308 nm LED phototherapy, **(F)** after 308 nm LED phototherapy, **(G)** before 308 nm excimer laser phototherapy, **(H)** after 308 nm excimer laser phototherapy.

Among the 10 patients who completed the treatment, the mean baseline LPSI scores were 7.3 ± 1.6 (LED side) and 7.3 ± 2.2 (excimer side) (*p* > 0.05). After treatment, the LPSI scores were 3.2 ± 2.9 (LED side) and 2.8 ± 3.0 (excimer side) (*p* > 0.05). There was a significant decrease in LPSI scores after treatment (*p* < 0.01) (Table 1). There were no significant differences observed in the reduction of LPSI scores, as well as erythema, infiltration, and scales scores between the two treatment sides, all *p* > 0.05 (Figure 1). Among the patients, 7 out of 10 achieved LPSI 50 on the side treated with 308 nm LED light, and 8 out of 10 achieved LPSI 50 on the side treated with excimer laser, *p* > 0.05. Additionally, 2 out of 10 patients achieved LPSI 100 on the 308 nm LED light-treated side and the excimer light-treated side. Both sides had a statistically significant difference in reduction in LPSI scores, both *p* < 0.01 (Table 1). The typical patient’s treatment images are shown in the Figure 2. Among the 12 patients who did not complete the study, 3 out of 12 achieved an LPSI score of 0/1, and 2 out of 12 reached an LPSI 50 response after 7–16 sessions. However, 5 out of 12 patients, showed minimal improvement or even experienced worsening of disease despite they have received 10–16 treatments (Supplementary Table S3).

**Table 1:** Patient local psoriasis severity index (LPSI) scores before and after treatment of 308 nm light emitting diode (LED) light versus 308 nm excimer laser.

Patient	Sex	Age	Fitzpatrick phototype	Lesion localization	308 nm excimer laser			308 nm LED light		
					Baseline LPSI	12 weeks LPSI	Response	Baseline LPSI	12 weeks LPSI	Response
1	M	55	III	Gluteal	9	0	LPSI 100	9	0	LPSI 100
2	M	28	III	Lower leg extension	6	0	LPSI 100	5	0	LPSI 100
3	F	47	III	Elbow extension	10	1	LPSI 90	9	1	LPSI 75
4	F	50	III	Trunk	8	2	LPSI 75	8	2	LPSI 75
5	F	69	III	Lower leg extension	4	1	LPSI 75	5	2	LPSI 50
6	M	32	III	Knee extension	6	3	LPSI 50	6	3	LPSI 50
7	F	65	IV	Trunk	8	3	LPSI 50	8	4	LPSI 50
8	F	28	III	Lower leg extension	4	2	LPSI 50	7	5	Non-responder
9	F	40	III	Lower leg extension	10	7	Non-responder	9	6	Non-responder
10	F	62	III	Lower leg extension	8	9	Non-responder	7	9	Non-responder
Total					7.3 ± 2.2	2.8 ± 3.0	*p* < 0.01	7.3 ± 1.6	3.2 ± 2.9	*p* < 0.01

### 3.3. Dermoscopic evaluation

According to dermatoscopic evaluation of key dermatoscopic features, an improvement was observed in both treatment sides compared to baseline. In our results, 80% background erythema was improved on both sides, 77.8% (excimer side) and 75.0% (LED side) of scales was improved, and all hemorrhagic spots and hemorrhagic crusts were improved slightly or markedly, all *p* > 0.05 (Table 2). The improvement of dermatoscopic features on the two sides was similar (Figure 2).

**Table 2:** Dermoscopic features of psoriatic plaques before and after treatment of 308 nm light emitting diode (LED) light versus 308 nm excimer laser.

Dermoscopic features	308 nm excimer laser			308 nm LED light		
	Baseline	12 weeks improved (slight or marked)	12 weeks unimproved	Baseline	12 weeks improved (slight or marked)	12 weeks unimproved
Background erythema, *n* (%)	10 (100)	8 (80)	2 (20)	10 (100)	8 (80)	2 (20)
Scales, *n* (%)	9 (90)	7 (77.8)	2 (22.2)	8 (80)	6 (75)	2 (25)
Hemorrhagic spots, *n* (%)	4 (40)	4 (100)	0 (0)	3 (30)	3 (100)	0 (0)
Hemorrhagic crusts, *n* (%)	1 (10)	1 (100)	0 (0)	1 (10)	1 (100)	0 (0)

### 3.4. Safety

Painful erythema was observed in 1 out of 22 patients. However, no serious adverse events were reported throughout the 12 weeks study period.

## 4. Discussion

Phototherapy was found in natural sunlight, serves as a reasonable and effective treatment with few side effects (1), it usually takes only a few minutes but the patients need to travel twice or thrice a week and waste time to wait before start of phototherapy (5, 8). Larko and Swanbeck first reported home phototherapy for patients with psoriasis in 1979 (9). Kemeny et al. (10) discovered that UVB-LED (311 nm) hold significant potential as a targeted phototherapy technology for psoriasis. This LED device offers high effectiveness, exceptional safety, portability, wearability, and this breakthrough may pave the way for the development of advanced home-based phototherapy devices in the future. The application of the 308 nm excimer laser for the treatment of psoriasis was initially introduced in 1997 (11), which gives photobiological effects theoretically superior to those provided by NB-UVB (3). Compared with NB-UVB, 308 nm excimer laser effectively treats resistant and localized psoriasis lesions with fewer treatment sessions and a reduced cumulative dosage (6). However, maintenance costs of 308 nm excimer laser remain quite expensive (12). Though much cheaper excimer light systems are on the market, compared to 308 nm LED light, its cost still relatively high. 308 nm LED home phototherapy seems to be a feasible option for treating local plaque psoriasis, in order to seek the possibility of safe, effective, and more convenient treatment for local plaque psoriasis. In theory, under the same wavelength, the penetration depth of LED light and excimer laser is expected to be similar. However, due to differences in the light source and emission methods, the light transmission and tissue absorption characteristics may vary slightly. So, we conducted a comparative study to evaluate whether there were any differences in efficacy and safety between the two lights.

In our study, we observed that the efficacy of 308 nm LED light was comparable to that of 308 nm excimer laser, with similar safety profiles. Objective assessments conducted by physicians using the LPSI scores showed similar treatment responses in the evaluated skin lesions. The LPSI reduction was found to be 62.0% on the excimer side and 55.7% on the LED side, indicating significant improvement in both sides. Dermoscopy can provide detailed visualization of key dermatoscopic features, allowing for the identification of subtle changes, and provides a more accurate evaluation of the treatment response (13). Changes in the vessel patterns were reported to be a predictor of the response of psoriatic patients to phototherapy (14). In both treatment sides, we observed similar vascular changes, implying a comparable mechanism of action for both types of light. Our findings suggest that 308 nm LED light treatment presents a viable alternative to 308 nm excimer laser.

According to previous publications, it has been demonstrated that 308 nm excimer therapy can upregulate Treg levels, indicating its potential contribution to immunological homeostasis (15). There is no cure for psoriasis and the objective of therapeutic interventions is to attain remission. Therefore, it is important for physicians to be mindful of reducing the burden on patients. In our study, more than half of the patients were unable to adhere to 12 weeks of treatment. In addition to poor treatment efficacy caused discontinue, 5 patients who achieved an improvement also had treatment interruption. During further patient inquiries, 4 patients provided feedback indicating that the hospital visit process was overly burdensome. It means frequent clinic visits were burdensome for these patients with localized involvement. It is worth noting that among the 5 patients who discontinued treatment due to poor treatment efficacy, 4 patients experienced localized expansion or spreading of the lesions. The poor efficacy maybe because of changes in activity of psoriasis disease. This highlights the importance of conducting a careful evaluation to determine if patients are in a stable phase before initiating localized phototherapy. Furthermore, it is important to monitor and reevaluation of patients’ disease activity throughout the course of treatment to make timely adjustments and ensure optimal treatment outcomes.

The aforementioned findings confirmed that 308 nm LED light has good therapeutic effect on local psoriasis, indicating a potential prospect of utilizing 308 nm LED light in home phototherapy as a cost-effective alternative to the currently available 308 nm excimer light for more affordable price. Furthermore, the implementation of home phototherapy has the potential to reduce the need for prescribing systemic and biologic therapies (9). Patients treated at home were reported to have a similar efficacy and a more positive evaluation of their treatment compared to patients treated in the outpatient setting (*p* < 0.001) (16). Previous studies have also demonstrated that in the hospital-based treatment group, eight patients discontinued the study due to missing more than 10 treatments. In contrast, none of the patients in the home-based treatment group missed a significant number of treatments (8). Home-based phototherapy may enhance treatment adherence among patients. However, our study was conducted in a hospital setting, it confirmed that the efficacy and safety of 308 nm LED light therapy were comparable to excimer laser therapy. However, further investigation is needed to assess the efficacy and safety of patients self-administering the treatment at home throughout the entire treatment period. Notably, patients require training before initiating home phototherapy. This training aims to provide them with the necessary knowledge and skills to ensure their safety and treatment efficacy (17).

This study has several limitations. Firstly, the limited sample size restricted the statistical analysis and influenced the conclusion drawn. Secondly, the study did not evaluate the long-term efficacy. Thirdly, this study assessed severity of target skin lesions, but did not evaluate the impact on severity of the whole body. Additionally, psoriasis is an autoinflammatory disease in which circulating and tissue cytokines play a significant role, as a result, treating one half of the body may inadvertently impact the other half, which may have a potential influence on results. Owing to the distinctive design of our study, we did not incorporate a conventional placebo group, which might have introduced potential placebo-related comfort effects. Further studies are needed to investigate the efficacy and implications of 308 nm LED light.

## 5. Conclusion

In conclusion, our study demonstrates that 308 nm LED light can serve as an alternative to 308 nm excimer laser therapy for the treatment of localized psoriasis. In office-based settings, the utilization of 308 nm LED light offers several advantages, including reduced equipment costs and a longer lamp lifespan, which in turn reduces the need for frequent replacements. However, perhaps the most significant benefit lies in its potential for home-based treatment, enabling localized patients to avoid frequent hospital visits and providing a more cost-effective option.

## Data availability statement

The raw data supporting the conclusions of this article will be made available by the authors, without undue reservation.

## Ethics statement

The studies involving humans were approved by the Ethics Committee of Shanghai Skin Disease Hospital (approval #2020-32). The studies were conducted in accordance with the local legislation and institutional requirements. The participants provided their written informed consent to participate in this study. Written informed consent was obtained from the individual(s) for the publication of any potentially identifiable images or data included in this article.

## Author contributions

YY: Writing – original draft, Writing – review & editing. JL: Writing – review & editing. YZ: Writing – review & editing. YS: Writing – review & editing.
