# Supplementary figures and images for: A prospective randomized half-body study: 308 nm LED light vs. 308 nm excimer laser for localized psoriasis
Source: Front Med (Lausanne). 2023 Nov 6;10:1275912. doi: 10.3389/fmed.2023.1275912 (PMC10657802; doi:10.3389/fmed.2023.1275912)

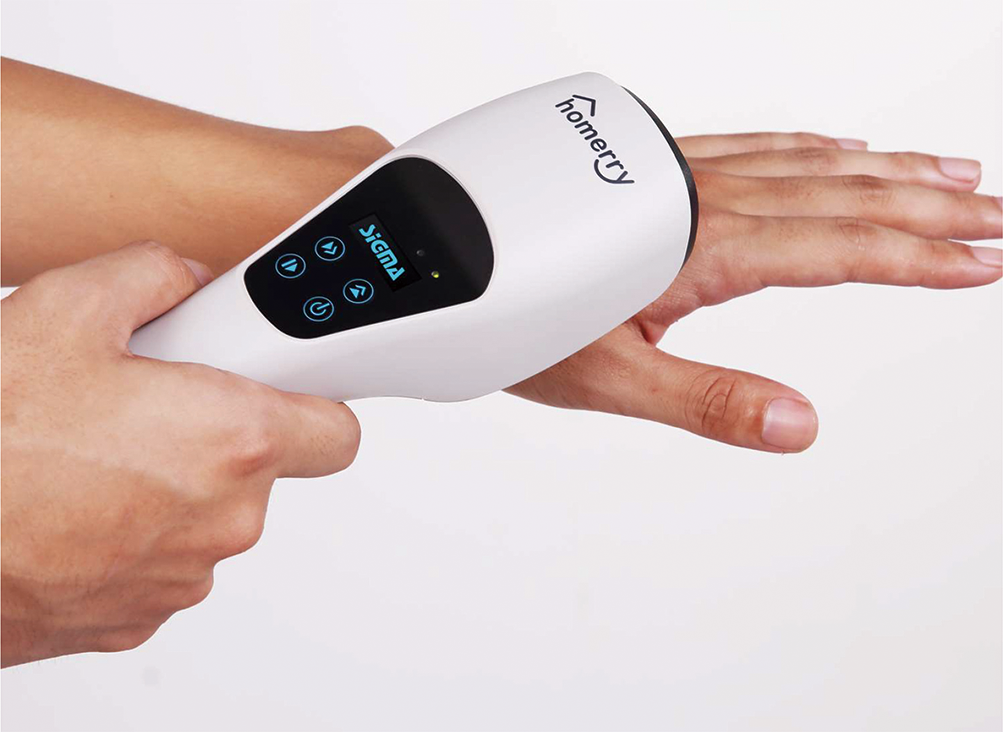

Supplement: Supplementary file 1 [file Image_1.TIF]
