# Supplementary material for: A prospective randomized half-body study: 308 nm LED light vs. 308 nm excimer laser for localized psoriasis
Source: Front Med (Lausanne). 2023 Nov 6;10:1275912. doi: 10.3389/fmed.2023.1275912 (PMC10657802; doi:10.3389/fmed.2023.1275912)
Supplement: Supplementary file 2 [file Table_1.docx]

Supplementary Material

Table S1. Inclusion and exclusion criteria.

| Inclusion criteria | Exclusion criteria |
| --- | --- |
| Adult patients (age ≥ 18) | Other types of psoriasis or other skin diseases that may influence disease assessment |
| Stable (≥6 months) mild-to-moderate localized psoriasis (PASI ≤ 10, BSA ≤ 10%, LPSI ≥ 4), with an equal distribution of left and right discrimination | Pregnant or breastfeeding woman |
| Without systemic treatment (acitretin, cyclosporine, methotrexate, phototherapy, biologics) in the last one month | With severe comorbidities |
| Without external drug treatment (except moisturizing cream) in the last two weeks | Other conditions not suitable for the study |
| Written informed consent was obtained |  |

Table S2. Dosing protocol.

| Initial dose for psoriasis | | | | | | | |
| --- | --- | --- | --- | --- | --- | --- | --- |
| Plaque thickness | | Induration score | | Fitzpatrick skin type I-III (dose in mJ/cm^2^) | | Fitzpatrick skin type IV-VI (dose in mJ/cm^2^) | |
| None | | 0 | | 0 | | 0 | |
| Mild | | 1 | | 500 | | 400 | |
| Moderate | | 2 | | 500 | | 600 | |
| Severe | | 3 | | 700 | | 900 | |
| Dose for subsequent treatments | | | | | | | |
| No effect | Minimal effect | | Good effect | | Considerable improvement | | Moderate/severe erythema (with or without blistering) |
| No erythema at12-24 h and no plaque improvement | Slight erythema at 12-24 h but no significant improvement | | Mild-to-moderate erythema response 12-24 h | | Significant improvement with plaque thinning or reduced scaliness or pigmentation occurred | |  |
| Typical dosing change from prior treatment dose | | | | | | | |
| Increase dose by 25% | Increase dose by 15% | | Maintain dose | | Maintain dose or reduce by 15% | | Reduce dose by 25% (treat around blistered area, do not treat blistered area until it heals, or crust disappears) |

| Patient | Sex | Age | Fitzpatrick phototypes | Lesion localizations | Total sessions | Withdraw reasons | 308-nm Excimer Laser | | 308-nm LED Light | |
| --- | --- | --- | --- | --- | --- | --- | --- | --- | --- | --- |
|  |  |  |  |  |  |  | Baseline LPSI | Last record LPSI | Baseline LPSI | Last record LPSI |
| 1 | M | 55 | III | Lower leg extension | 16 | Improved | 9 | 1 | 8 | 1 |
| 2 | F | 49 | III | Elbow extension | 14 | Improved | 8 | 1 | 8 | 1 |
| 3 | F | 74 | III | Lower leg Flexion | 8 | Improved | 6 | 0 | 6 | 1 |
| 4 | M | 33 | III | Lower leg extension | 9 | Improved | 9 | 4 | 9 | 4 |
| 5 | M | 26 | III | Lower leg extension | 7 | Improved | 10 | 5 | 8 | 2 |
| 6 | M | 51 | III | Trunk | 14 | Poor efficacy | 8 | 6 | 7 | 7 |
| 7 | M | 65 | III | Elbow extension | 13 | Poor efficacy | 7 | 10 | 7 | 10 |
| 8 | M | 28 | IV | Lower leg extension | 10 | Poor efficacy | 8 | 10 | 7 | 8 |
| 9 | M | 64 | IV | Lower leg extension | 10 | Poor efficacy | 8 | 10 | 7 | 10 |
| 10 | 1 | 28 | III | Lower leg extension | 16 | Poor efficacy | 9 | 12 | 9 | 11 |
| 11 | F | 28 | III | Forearm extension | 1 | Adverse events | 4 | 4 | 4 | 4 |
| 12 | M | 31 | III | Lower leg extension | 1 | personal reasons | 9 | 9 | 8 | 8 |

Table S3. Withdraw patients’ Local Psoriasis Severity Index (LPSI) scores before and after treatment of 308-nm LED Light versus 308-nm Excimer Laser.
